# Formation of aroma compounds in smoked chickens during the sugar-smoking process using SPME/SAFE-GC–MS and ESR technologies

**DOI:** 10.1016/j.fochx.2025.102646

**Published:** 2025-06-12

**Authors:** Runmi Tao, Teng Liu, Huan Liu, Jun Qi, Kexin Cheng, Yumin Niu, Yuting Wang, Yingying Zhang, Dengyong Liu

**Affiliations:** a College of Food Science and Technology, Bohai University, Jinzhou 121013, China; b School of Food Engineering, Ludong University, Yantai 264025, China; c Anhui Engineering Laboratory for Agro-products Processing, College of Tea & Food Science and Technology*,* Anhui Agricultural University*,* Hefei 230036*,* China

**Keywords:** Sugar-smoked chicken, Aroma compounds, Lipid oxidation, Free radicals

## Abstract

Free radicals play crucial roles in the development of grilled meat flavors, but the mechanisms by which they contribute to the formation of flavor compounds in smoked chickens remain unclear. In this study, GC–MS, ESR, and UPLC techniques were employed to analyze the dynamic changes in key flavor compounds, sugars, and free radicals in smoked chickens. A total of 22 (OAVs >1) key volatile compounds were identified in smoked chickens. Additionally, the concentrations of hydroxyl radicals, alkyl peroxy radicals, alkyl radicals, and alkoxy radicals significantly increased with prolonged heating. These free radicals were positively correlated with the formation of flavor compounds such as furfural, heptanal, and (*E*,*E*)-2,4-decadienal, whereas they were negatively correlated with the formation of butanal, pentanal, and hexanal. This research provides valuable insights for the precise control and optimization of flavors in sugar-cured meat products.

## Highlights

• Alkoxy radicals, alkyl radicals, alkyl peroxyl radicals and hydroxyl radicals were detected in smoked chickens.
• A total of 22 key aroma compounds were detected.
• Alkoxy radicals are the most important free radicals for lipid oxidative degradation in smoked chickens.
• Radicals play a dual role in the formation and degradation of flavor compounds.

## 1 Introduction

The sugar-smoked meat products are popular among consumers due to their unique flavor. The regulation and enhancement of flavor quality is a direct way to meet the demands of consumers for a better life (Chang et al., 2021). The sugar-smoked meat products are prepared using traditional smoking methods, where sucrose is heated at high temperatures to produce a rich and distinctive smoky aroma to the product (Wang et al., 2022). Compared to the traditional wood smoking method and tea smoking method, the sugar smoking method results in a softer and more rounded aroma (Zhang et al., 2022). This smoky aroma mainly comes from furfural substances formed by sucrose cracking. Under high-temperature conditions, the pyrolysis of sucrose to produce furfural involves the cleavage and recombination of chemical bonds within the molecule, and the breaking of some chemical bonds leads to the redistribution of electrons, resulting in the generation of free radicals, such as alkyl radicals and alkoxy radicals (Zhou et al., 2023). Additionally, the pyrolysis of sucrose produces glyceraldehyde, which is necessary for the formation of free radicals (Cämmerer et al., 1999). The homolytic and heterolytic cleavage reactions of sucrose during pyrolysis can also produce free radicals, including H^+^, O^−^ and O^2−^ (Gu et al., 2012). Free radicals are highly reactive and can attack other compounds. For example, when heated at 85 °C and 100 °C, free radicals can initiate oxidation reactions in polyunsaturated fatty acids, leading to the formation of dienal compounds, which are key fatty aroma compounds in meat products (Huang et al., 2021). Bao et al. (2020) measured the content of free radicals formed in meat with different raw materials after roasting at 200 °C for 20 min and found that chicken thighs exhibited the highest content. In summary, it is hypothesized that the pyrolysis of sucrose during the sugar-smoking process generates a substantial number of free radicals. These free radicals are adsorbed onto the surface of the muscle tissue and subsequently penetrate into the inner layers, thereby facilitating the formation of flavor compounds. However, there remains a lack of systematic research on the changes in sugar types and free radicals in sugar-smoked chicken over different smoking durations, as well as their relationships with the aroma compounds in roasted meat.

Electron spin resonance (ESR) technology is a modern analytical method that allows for the rapid, simple, and direct detection of compounds containing unpaired electrons. For example, ESR has been extensively applied to measure the free radical content in foods (Hashim et al., 2024). Consequently, ESR is an essential technique for directly identifying the types and quantifying the content of free radicals in smoked chicken.

The selection of an appropriate extraction method plays a crucial role in the identification and quantification of aroma compounds in food products. Solid-phase microextraction (SPME) is one of the most widely applied techniques in food flavor analysis. Similarly, the solvent-assisted flavor evaporation (SAFE) method offers a gentle and comprehensive extraction approach, ensuring high accuracy and satisfactory detection limits under low-temperature and low-pressure conditions. Gas chromatography-mass spectrometry (GC–MS) remains a standard analytical tool for detecting and characterizing volatile compounds in complex, unknown samples. For instance, Zhang et al. (2021) employed both SPME and SAFE to analyze flavor changes in cooked mutton meatballs during storage. These findings demonstrated that while SPME extracted a broader range of flavor compounds, SAFE achieved a higher extraction efficiency, emphasizing the necessity of integrating multiple methods for comprehensive aroma profiling. In conclusion, the combination of SPME and SAFE with GC–MS offers an accurate and comprehensive approach for identifying key aroma in smoked chicken.

Therefore, this study aimed to (1) analyze the types of sugars and their degradation mechanisms in smoked chickens subjected to varying heating times using the UPLC method, (2) employ SPME/SAFE-GC–MS and retention index identification techniques to determine the molecular structures of volatile compounds, (3) utilize ESR technology to examine the content and evolution of free radicals in smoked chickens, and (4) conduct correlation analysis to elucidate the relationships among sugars, free radicals, and the formation of key aroma compounds. The findings of this study offer a solid theoretical foundation for the continued research and development of sugar-smoked meat products, with a particular focus on precise aroma control and the enhancement of product quality.

## 2 Materials and methods

### 2.1 Materials

The following compounds were used in this research: anhydrous ethanol (chromatographic purity) (Shandong West Asia Chemical Industry Co., Ltd., Shandong, China), methanol (chromatographic purity), dichloromethane (chromatographic purity) (Sinopharm Chemical Reagent Co., Ltd., Shanghai, China). Flavor standards: 2-methyl-3-heptanal (99 %), butyraldehyde (99 %), glutaraldehyde (98 %), hexanal (99 %), furfural (99 %), heptanal (99 %), 1-(2-furanyl)-ethenone (99 %), 5-methylfurfural (99 %), 1-octen-3-one (98 %), 1-octen-3-ol (98 %), octanal (98 %), (*E*)-2-octenal (96 %), fury hydroxymethyl ketone (96 %), nonanal (99 %), (*E,E*)-2,6-nonadienal (99 %), (*E,Z*)-2,6-nonadienal (99 %), (*E*)-2-nonenal (97 %), decanal (99 %), (*E,E*)-2,4-nonadienal (94 %), (*E*)-2-decenal (99 %), undecanal (98 %), (*E,E*)-2,4-decadienal (94 %), tetradecimal (98 %), 5,5-dimethyl-1-pyrroline-N-oxide (DMPO) (Aladdin Shanghai Jingpure Biochemical Technology Co., Ltd., Shanghai, China). C_7_-C_40_ n-alkane labels (Supelco, USA).

### 2.2 Sample preparation

The fresh chicken thighs (120.00 ± 5.00 g) used in this study were purchased from Wanwei Supermarket in Jinzhou, China. The chicken breed was sanhuang chicken (with a ketone weight of 500.00 ± 50.00 g). The experiment included two treatment groups, the group without smoking material (Group W) and the sugar-smoked group (Group X), which were maintained under identical conditions.

First, the chicken thighs were washed and cooked in a solution of 5 % saline water in an induction cooker (Midea WT2118, Tianjin, China) at a mass ratio of 1:2. The water was brought to a boil and maintained at that temperature for 30 min. After boiling, the thighs were drained of any excess water and stored for later use. The chicken thighs that were only boiled without being smoked were labeled as W0. The sugar-smoked group was subjected to smoking treatment with sucrose as the primary smoking material. Each group was weighed with 24 g of sucrose and smoked using a device made in our laboratory, this device consisted of a heated plate (C-MAG HP10 IKA electric heating plate, IKA Group, Germany), a multichannel temperature indicator, and a stainless-steel iron pot (Supor SZ30T1, Wuhan, China). After the temperature at the center of the bottom of the pot reached 400 °C, the cooked chicken thighs were placed to start smoking the samples. The group without smoking material was heated under the same conditions in the same apparatus. The samples in the sugar-smoked group were labeled X1, X2, X3, X4, and X5, representing the samples produced by sugar smoking for 1 min, 2 min, 3 min, 4 min, and 5 min, respectively. Similarly, the group without smoking material was labeled W1, W2, W3, W4, and W5. Three chicken thighs from each treatment group were heated five times (*n* = 30). The chicken thighs were deboned and pulverized into a powder using liquid nitrogen. This powder was stored at −80 °C and measured within one week.

### 2.3 Determination of the sugar concentration in sugar-smoked chicken

The determination of sugar content was conducted with slight modifications based on the method described by Castellane et al. (2014). First, 5 g of sample was accurately weighed into a centrifuge tube, followed by the addition of 25 mL of water. The mixture was vortexed thoroughly and subjected to ultrasonic treatment for 30 min. Subsequently, 2 mL each of zinc sulfate solution and potassium ferrocyanide solution were added as protein precipitants. After thorough mixing, the sample was centrifuged at 4000 ×*g* for 5 min. The resulting supernatant was transferred to a 50 mL volumetric flask. The extraction procedure was repeated once, and the two supernatants were combined, brought to volume with water, and mixed well for subsequent derivatization. Appropriate amounts of fructose, sucrose, glucose, and ribose standard solutions were accurately weighed and dissolved in water. The resulting solutions were then transferred to 50 mL volumetric flasks, and the volume was adjusted to the mark with water, thereby preparing working standard solutions with concentrations of 1.18 mg/mL, 1.01 mg/mL, 1.05 mg/mL, and 1.10 mg/mL, respectively.

To 0.5 mL of the derivatization solution and 0.5 mL of the sugar mixture working solution, 0.5 mL of 0.5 mol/L 1-phenyl-3-methyl-5-pyrazolone (PMP) methanol solution and 0.2 mL of 0.5 mol/L sodium hydroxide solution were added in a sealed test tube. The mixture was thoroughly mixed and incubated in a 70 °C water bath for 1 h. After cooling to ambient temperature, 0.2 mL of 0.5 mol/L hydrochloric acid solution was added and mixed. Subsequently, 1 mL of trichloromethane was injected into the mixture, which was mixed vigorously for 1 min. The sample was centrifuged at 3000 *g* for 5 min, and the lower trichloromethane layer was discarded. The aqueous phase was extracted twice with trichloromethane to remove excess PMP. Then, the resulting supernatant was filtered through a 0.22 μm membrane. Finally, the filtrate was tested using HPLC (LC-2030C 3D PLUS, Shimadzu Corp., Tokyo, Japan).

The mobile phase consisted of potassium dihydrogen phosphate solution (0.1 mol/L, pH 6.85) and acetonitrile at a ratio of 82:18. A Waters Symmetry C18 column (4.6 mm × 250 mm × 5 μm, Shimadzu Corp., Tokyo, Japan) was used for separation. The gradient elution conditions were as follows: 0–22 min, 18 % acetonitrile; 22.01–30 min, 18–40 % acetonitrile; 30.01–32 min, 40 % acetonitrile; and 32.01–35 min, 40–18 % acetonitrile. The flow rate was set at 1.0 mL/min, the injection volume was 10 μL, the column temperature was 25 °C, and the detection wavelength was 250 nm.

### 2.4 Extraction of aroma compounds from sugar-smoked chickens

#### 2.4.1 Extraction of aroma compounds using headspace solid-phase microextraction (HS-SPME)

In accordance with the methodology described by Zhang et al. (2022), 4.00 g of the sample was placed in a 20 mL headspace vial (Ningbo Hamai Instrument Science and Technology Co., Ltd., Ningbo, China), and 6 μL of 2-methyl-3-heptanone (0.812 μg/μL) was added, followed by immediate sealing of the vial with a polytetrafluoroethylene (PTFE) rubber sheet. After the vials were allowed to equilibrate at 40 °C for 15 min, they were adsorbed using a 75 μm CAR/PDMS fiber at 40 °C for 45 min. Finally, the extraction head was inserted into the gas chromatography inlet and desorbed at 250 °C for 5 min.

#### 2.4.2 Extraction of aroma compounds by solvent-assisted flavor evaporation (SAFE)

Following the method of Liu et al. (2022), 80 g of the sample was placed into a 250 mL conical flask, and 150 mL of chromatography-grade dichloromethane and 20 μL of internal standard (917 mg/L 2,4,6-trimethylpyridine) were added. The mixture was vibrated at 200 rpm on a magnetic stirrer for 2 h, and the dichloromethane layer was collected by decanting. The extraction of the lower phase was repeated two more times with 100 mL and 50 mL of chromatography-grade dichloromethane. Finally, the extract was mixed with chromatography-grade dichloromethane and adjusted to a volume of 300 mL. SAFE of the volatiles was conducted at 40 °C under high vacuum (10^−4^ Pa). The collected fractions were dehydrated overnight with anhydrous sodium sulfate. After separation, the extract was concentrated to 2.5 mL in a Vigreux distillation unit (Yang et al., 2018). The concentrate was further concentrated to 0.5 mL under a gentle nitrogen stream.

#### 2.4.3 Detection of aroma compounds in smoked chickens

Following the method described by Jiang et al. (2022), the volatile compounds in the samples were qualitatively and quantitatively analyzed by gas chromatography–mass spectrometry (GC–MS) (Shimadzu Corp., Tokyo, Japan), an Rtx-5MS capillary column (30 m × 0.25 mm × 0.25 μm) was used for the analysis. The carrier gas flow rate was set at 1 mL/min, and the splitless injection mode was used. The initial oven temperature was set as follows: 40 °C for 4 min, increased to 100 °C at a rate of 4 °C/min for 5 min, and finally increased to 200 °C at a rate of 4 °C/min for 10 min. The ion source temperature was set at 230 °C, and the selective ion monitoring/scanning mode ranged from 30 to 500 *m*/*z*.

### 2.5 Identification, quantitation, and OAV analysis of aroma compounds in sugar-smoked chickens

For the identification analysis, comparisons were made with experimental compounds based on the retention times of the n-alkanes (C_7_-C_40_). Aroma compounds were identified by searching the mass spectrometry database (NIST). A quantitative method based on the relationship between the peak area and concentration of internal standards was used to estimate the content of volatile flavor compounds. First, volatile flavor compounds with odor activity values (OAVs) > 1 were selected based on the quantification results of internal standards. Next, flavor standards with concentrations ranging from 1 ng/g to 10 mg/g were added to the smoked chicken matrix and adsorbed according to the extraction conditions of the samples. The volatile flavor standards were detected using GC–MS. On the basis of these results, a standard curve showing the relationship between the concentration of volatile flavoring substances and the peak area was established. To ensure that the results were accurate, the entire experimental procedure was repeated six times, and the final results were expressed as ng content per gram of smoked chicken.

OAVs can effectively reflect the degree to which volatile flavoring substances contribute to the overall flavor of smoked chickens. When the OAVs are <1, the concentration of the flavor substance is below the threshold that humans can perceive, and the contribution of the substance to the overall flavor is relatively small. In contrast, when the OAVs are ≥1, the flavoring substance contributes more to the overall flavor of the smoked chickens (Grosch, 1994). The formula for calculating OAVs is as follows:

$$\text{OAVs}=C_{i}/T_{i}$$

where:

C_i_ - concentration of the flavor substance to be tested/(ng/g);

T_i_ - the sensory threshold of the flavor substance to be tested/(ng/g).

### 2.6 Determination of free radical concentrations in sugar-smoked chicken

Owing to the extremely short life cycle of reactive radicals, which usually lasts for a few milliseconds to microseconds, and extremely low concentrations, their direct detection is difficult or impossible. Spin-trapping agents are added to free radicals to obtain more stable free radicals before detection. The determination of free radicals was conducted with slight modifications to the method described by Yang et al. (2022), utilizing a Bruker A300 ESR Spectrometer (Bruker, Germany). The experimental parameters were set as follows: 3510 G (center field), 100 G (sweep width), 3460 G (static field), 1024 points (resolution), 9.85 GHz (microwave frequency), 19.71 mW (microwave power), 1.0 G (modulation amplitude), 100 kHz (modulation frequency), 60 ms (conversion time), 10.24 ms (time constant), 61.44 s (sweep time), and 2 accumulated scans. To 1 mL of solvent, 1 mg of sample and 10 ppm DMPO (trapping agent) were added, and the contents were transferred to a thin-walled cylindrical sample tube made of high-purity quartz. The DPPH standard sample with a known g factor was tested on the machine at the same time as the sample, and the g value of the sample was obtained using the comparison method. The experimental spectra of the samples were fitted using the spin fitting function in Bruker Xenon software. Based on the hyperfine coupling constants of the DMPO-radical adducts obtained from the fitting, the types of free radicals were determined. Quantification of the free radicals was performed by measuring the peak areas of different free radicals in the fitted spectrum. Eq. (2-1) shows that gx = gsBs/Bx. The test spin concentration was determined via the comparative method, where the standard sample was compared with an unknown sample. In the experiment, the test conditions were consistent, and the spin concentration was calculated using Equation Eqs. (2-2).

(2-1) V=gsβBs/h=gxβBx/h

(2-2) $$\mathrm{Nx}=\left(\text{AxGs}/\text{AsGx}\right)\mathrm{Ns}$$

Here, N indicates the spin number of the sample, A indicates the area of integration of the spectral line, G indicates the test magnification, and the subscripts s and x indicate the standard sample and the sample to be tested, respectively.

The nature of the standard sample and the sample to be tested must be similar to ensure that the measurement at the microwave power is not in a saturated state. To eliminate the differences between the two cavities of the dual-sample cavity, two cavities of the error caused by the standard sample and the unknown sample were measured once, the two exchange positions were measured again, and the average of the two measurements was used for the calculation.

### 2.7 Statistical analysis

Statistical analysis was performed using SPSS 23.0 software, with a one-way ANOVA conducted to assess significant differences (*p* < 0.05). The results are expressed as the means ±standard deviations (*n* = 3). The data were visualized, and graphs were plotted using the Origin 2019 software. To ensure the reliability of the results, each test had three replicates.

## 3 Results and discussion

### 3.1 Changes in sugar concentrations in smoked chickens during the sugar-smoking process

Sucrose, which is used as the only smoking material for smoked chickens, undergoes cleavage at its glycosidic bond (fructosyl‑oxygen bond) at 400 °C, releasing free glucose and fructose. The continued pyrolysis of fructose and glucose is the primary source of furan compounds, which impart a characteristic sugar-smoked flavor to chickens (Liu et al., 2024). Glucose, ribose, fructose, and sucrose serve as important flavor precursors in various food products.

The contents of the four sugars detected in the smoked chickens are shown in Table 1. First, the sucrose content in sugar-smoked chicken increases rapidly with extended smoking time, followed by a decline. This phenomenon may be attributed to the evaporation of moisture from the chicken due to the high temperature in the early stages of smoking. Concurrently, sucrose may begin to dissolve and penetrate into the meat tissues, resulting in an initial increase in sucrose content. As the smoking time increased, sucrose underwent a caramelization reaction to produce glucose, fructose, and other sucrose derivatives that adsorbed onto the chickens, decreasing the sucrose content. In contrast, the glucose content in chicken increased significantly (*P* < 0.05) after sugar smoking compared with that in the group without smoking material, which was attributed to the high-temperature cracking of sucrose to produce glucose. The non-significant change in glucose content occurred after 2 min in the sugar-smoked group with increasing sugar-smoking time because the glucose content that was continuously enriched in the chickens might have reached a dynamic equilibrium with the consumption of glucose (Patwardhan et al., 2009). The changes in the fructose content in the sugar-smoked group were similar to those in the glucose content, which gradually stabilized during the smoking process, reaching dynamic equilibrium. It is important to note that the fructose content is significantly higher than that of glucose, which may be attributed to the greater thermal stability of fructose's furanose ring structure compared to the pyranose ring structure of glucose. Furthermore, under high-temperature conditions, glucose can undergo isomerization to fructose, further increasing the fructose content. Ribose is the most important flavor precursor in chickens and the most reactive and least stable sugar. Ribose is dehydrated at a high temperature to form 2-furaldehyde and 2-furanmethanethiol (Liu et al., 2021). Aliani and Farmer (2005) reported increase significantly in the content of 2-furan methanethiols after the addition of ribose to chickens. The ribose content in both the sugar-smoked group and the group without smoking material decreased with increasing smoking time, and the rate of decrease in the ribose content in the group without smoking material was greater than that in the sugar-smoked group. This may be attributed to the high glucose content in the sugar-smoked group, which competitively inhibited the reaction of ribose with amino acids. (See Table 2.)

**Table 1:** Changes of sugar content in smoked chicken during the smoking process.

Smoking method	Time (min)	Glucose (mg/kg)	Ribose (mg/kg)	Fructose (mg/kg)	Sucrose (mg/kg)
Without smoking material	0	9.60 ± 0.51^a^	7.72 ± 0.05^a^	–	–
	1	8.70 ± 0.21^Ab^	6.56 ± 0.02^Ab^	–	–
	2	7.53 ± 0.42^Ac^	6.51 ± 0.14^Ab^	–	–
	3	4.85 ± 0.45^Ad^	5.89 ± 0.28^Ac^	–	–
	4	4.01 ± 0.64^Ae^	4.87 ± 0.17^Ad^	–	–
	5	3.58 ± 0.37^Ae^	4.86 ± 0.04^Ad^	–	–
Sugar-smoked	1	17.93 ± 0.52^Bb^	5.49 ± 0.07^Ba^	367.06 ± 24.27^a^	5811.59 ± 187.91^c^
	2	28.21 ± 3.10^Ba^	5.43 ± 0.27^Bab^	365.58 ± 78.71^a^	6048.96 ± 138.29^c^
	3	29.27 ± 1.19^Ba^	5.23 ± 0.1^Bab^	366.06 ± 68.46^a^	6794.25 ± 39.11^b^
	4	29.29 ± 1.68^Ba^	5.21 ± 0.05^Bb^	391.73 ± 38.86^a^	7208.80 ± 201.27^a^
	5	27.48 ± 1.92^Ba^	4.81 ± 0.1^Ac^	412.19 ± 37.05^a^	6745.89 ± 141.26^b^

Note: Lower case letters indicate significant differences between smoked chickens with different smoking times, and upper case letters indicate significant differences between different sugar-smoked groups with the same smoking time (*P* < 0.05).

**Table 2:** Changes in the content of volatile flavor compounds in sugar-smoked chicken thighs during the smoking process.

RI	CAS n	Compound	W0	W1	W2	W3	W4	W5	X1	X2	X3	X4	X5	Extraction method
Aldehyde														
607	123–72-8	Butyraldehyde	1.75 ± 0.17^c^	87.32 ± 4.27^b^	9.08 ± 2.06^c^	19.19 ± 0.17^c^	351.62 ± 9.46^a^	–	86.6 ± 2.30^b^	366.38 ± 22.27^a^	–	–	–	SPME
707	110–62-3	Valeraldehyde	192.88 ± 3.65^d^	344.23 ± 8.67^b^	370.65 ± 3.20^a^	340.06 ± 3.16^b^	352.15 ± 0.81^ab^	61.15 ± 2.39^f^	180.29 ± 1.89^f^	191.76 ± 6.8^d^	155.08 ± 0.82^e^	233.87 ± 14.91^c^	218.55 ± 5.28^c^	SPME
715	1576-87-0	(E)-2-Pentenal	0.33 ± 0.05^f^	0.99 ± 0.01^bcd^	1.18 ± 0.17^b^	0.62 ± 0.01^e^	0.74 ± 0.13^de^	1.24 ± 0.04^b^	–	0.62 ± 0.09^e^	0.87 ± 0.02^cde^	3.24 ± 0.03^a^	1.13 ± 0.18^bc^	SPME
806	66–25-1	Hexanal	2557.97 ± 55.89^d^	5345.82 ± 162.52^a^	5551.83 ± 231.58^a^	5562.33 ± 66.16^a^	5736.66 ± 184.42^a^	516.3 ± 66.07^e^	3370.98 ± 145.94^c^	3652.22 ± 46.05^bc^	4030.44 ± 25.53^b^	3255.46 ± 15.11^c^	3530.91 ± 161.89^c^	SPME/SAFE
814	505–57-7	2-Hexenal	1.48 ± 0.32^d^	5.91 ± 0.62^b^	8.3 ± 0.53^a^	0.28 ± 0.04^e^	–	6.36 ± 0.08^b^	2.49 ± 0.19^c^	1.43 ± 0.24^d^	–	–	–	SPME
814	6728-26-3	(E)-2-Hexenal	0.95 ± 0.06^e^	1.80 ± 0.35^de^	2.68 ± 0.10^d^	1.33 ± 0.01^e^	4.67 ± 0.05^c^	2.88 ± 0.20^d^	8.45 ± 1.04^a^	4.41 ± 0.02^c^	5.42 ± 0.03^bc^	6.42 ± 0.03^b^	4.48 ± 0.34^c^	SPME
873	1070–66-2	2-n-Butylacrylaldehyde	0.24 ± 0.04^e^	0.44 ± 0.04^de^	0.69 ± 0.10^de^	0.55 ± 0.08^de^	0.93 ± 0.04^de^	–	2.71 ± 0.17^d^	15.87 ± 0.83^c^	33.56 ± 1.90^a^	17.07 ± 0.90^c^	22.02 ± 0.07^b^	SPME
905	111–71-7	Heptanal	78.18 ± 1.32^g^	165.53 ± 1.79^c^	161.72 ± 10.34^c^	113.66 ± 5.18^f^	124.86 ± 4.15^ef^	26.21 ± 3.09^h^	135.4 ± 11.17^de^	243.86 ± 7.93^a^	148.25 ± 0.45^cd^	205.81 ± 3.65^b^	261.56 ± 9.36^a^	SPME/SAFE
913	6728-31-0	(Z)-4-Heptenal	1.16 ± 0.06^cd^	3.21 ± 0.23^b^	3.35 ± 0.07^b^	1.37 ± 0.02^cd^	2.98 ± 0.28^b^	1.48 ± 0.11^cd^	5.01 ± 0.29^a^	0.98 ± 0.05^d^	1.5 ± 0.01^c^	–	–	SPME
913	18,829–55-5	(E)-2-Heptenal	7.7 ± 0.99^ef^	18.68 ± 1.26^bc^	11.1 ± 0.86^de^	14.08 ± 1.32^cd^	20.45 ± 0.99^b^	27.87 ± 3.89^a^	12.03 ± 0.62^de^	4.39 ± 0.06^fg^	2.73 ± 0.35^g^	14.97 ± 0.95^cd^	–	SPME/SAFE
971	4313–03-5	(E,E)-2,4-Heptadienal	2.43 ± 0.11^efg^	4.38 ± 0.07^cdef^	6.57 ± 0.22^bcd^	3.8 ± 0.15^def^	6.68 ± 0.47^bcd^	7.45 ± 0.44^bc^	4.89 ± 0.16^bcde^	7.69 ± 0.01^b^	26.42 ± 3.06^a^	1.45 ± 0.02^fg^	–	SPME/SAFE
982	100–52-7	Benzaldehyde	12.26 ± 0.39^de^	13.32 ± 1.19^d^	10.85 ± 0.65^e^	17.26 ± 0.11^c^	23.15 ± 0.03^b^	25.74 ± 1.45^a^	–	–	–	–	–	SPME/SAFE
1005	124–13-0	Octanal	114.07 ± 2.54^f^	216.2 ± 2.65^bc^	251.95 ± 10.52^a^	201.04 ± 26.24^c^	239.57 ± 8.21^ab^	63.42 ± 0.78^g^	163.62 ± 0.06^d^	160.47 ± 2.39^d^	163.73 ± 1.87^d^	128.39 ± 6.00^ef^	154.7 ± 3.83^de^	SPME/SAFE
1013	2548-87-0	(E)-2-Octenal	32.88 ± 4.53^e^	9.61 ± 0.32^f^	18.15 ± 0.01^f^	9.37 ± 1.28^f^	10.72 ± 0.38^f^	260.69 ± 0.01^a^	80.28 ± 6.22^d^	35.59 ± 0.52^e^	209.55 ± 8.57^b^	254.19 ± 5.55^a^	137.39 ± 8.82^c^	SPME/SAFE
1020	90–02-8	2-Hydroxybenzaldehyde	–	–	–	–	–	–	–	–	–	21.93 ± 0.89^b^	67.82 ± 1.48^a^	SPME
1081	122–78-1	Phenylacetaldehyde	–	2.15 ± 0.16^c^	0.92 ± 0.15^e^	1.43 ± 0.11^d^	3.22 ± 0.01^b^	3.85 ± 0.42^a^	1.56 ± 0.10^d^	–	–	–	–	SPME/SAFE
1104	124–19-6	Nonanal	228.83 ± 7.16^f^	464.21 ± 9.14^cd^	566.27 ± 38.08^ab^	358.91 ± 13.40^e^	450.05 ± 7.88^d^	346.46 ± 0.96^e^	472.24 ± 8.53^cd^	588.14 ± 34.11^a^	461.78 ± 12.74^cd^	432.74 ± 18.55^d^	511.34 ± 1.33^bc^	SPME/SAFE
1112	18,829–56-6	(E)-2-Nonenal	21.21 ± 1.54^f^	48.47 ± 0.50^d^	66.58 ± 1.03^b^	38.68 ± 0.79^e^	39.58 ± 2.39^e^	57.16 ± 0.52^c^	42.78 ± 2.16^e^	63.1 ± 0.24^b^	61.34 ± 1.50^bc^	80.01 ± 3.57^a^	43.85 ± 0.61^de^	SPME/SAFE
1120	17,587–33-6	(E,E)-2,6-Nonadienal	0.84 ± 0.01^d^	7.51 ± 1.44^b^	–	1.81 ± 0.08^d^	–	4.99 ± 0.22^c^	1.08 ± 0.12^d^	3.86 ± 0.24^c^	10.78 ± 0.62^a^	4.11 ± 0.70^c^	–	SPME
1120	557–48-2	(E,Z)-2,6-Nonadienal	2.52 ± 0.03^d^	1.76 ± 0.24^d^	2.46 ± 0.42^d^	3.21 ± 0.81^d^	2.8 ± 0.19^d^	2.03 ± 0.08^d^	3.08 ± 0.11^d^	6.42 ± 0.49^c^	11.31 ± 0.60^a^	9.39 ± 1.28^b^	–	SPME
1120	5910-87-2	(E,E)-2,4-Nonadienal	10.96 ± 0.03^d^	27.42 ± 0.13^c^	36.58 ± 1.44^b^	21.11 ± 2.59^c^	21.73 ± 0.17^c^	23.48 ± 0.81^c^	25.9 ± 2.92^c^	47.41 ± 1.30^a^	48.43 ± 0.62^a^	47.3 ± 3.56^a^	50.14 ± 5.40^a^	SPME/SAFE
1204	112–31-2	Decanal	20.86 ± 0.86^f^	37.69 ± 0.26^d^	52.23 ± 0.33^a^	36.16 ± 1.19^d^	42.77 ± 0.40^bc^	37.18 ± 0.57^d^	38.26 ± 0.46^d^	40.84 ± 0.52^c^	44.42 ± 1.17^b^	41.05 ± 1.14^c^	33.29 ± 0.94^e^	SPME/SAFE
1212	2497-25-8	(Z)-2-Decenal	–	–	–	–	10.92 ± 0.14a	–	6.35 ± 0.71b	5.64 ± 0.09b	–	6.85 ± 1.34b	–	SAFE
1212	3913-81-3	(E)-2-Decenal	10.96 ± 0.03^g^	36.42 ± 1.36^de^	36.81 ± 0.96^de^	24.89 ± 2.08^f^	35.5 ± 0.01^e^	41.34 ± 0.06^c^	40.37 ± 1.03^cd^	41.21 ± 3.14^c^	43.99 ± 0.91^c^	58.06 ± 0.26^a^	52.15 ± 0.79^b^	SPME/SAFE
1220	25,152–83-4	(E,Z)-2,4-Decadienal	10.76 ± 0.53^g^	27.03 ± 0.63^d^	35.79 ± 1.88^c^	23.18 ± 1.49^e^	18.7 ± 1.17^f^	–	33.73 ± 1.44^c^	54.95 ± 0.57^b^	53.97 ± 0.28^b^	64.3 ± 0.09^a^	51.92 ± 0.37^b^	SPME/SAFE
1220	25,152–84-5	(E,E)-2,4-Decadienal	19.54 ± 0.14^h^	50.79 ± 1.04^e^	61.88 ± 0.86^d^	41.72 ± 2.7^f^	26.71 ± 0.50^g^	47.1 ± 1.55^e^	51.28 ± 1.47^e^	63.89 ± 1.61^d^	84.53 ± 3.38^b^	114.17 ± 0.89^a^	74.88 ± 0.86^c^	SPME/SAFE
1303	112–44-7	Undecanal	1.7 ± 0.03^f^	4.6 ± 0.01^de^	5.72 ± 1.22^de^	5.59 ± 0.46^de^	5.45 ± 0.03^de^	5.42 ± 0.03^de^	8.2 ± 0.94^bc^	3.83 ± 0.15^ef^	6.44 ± 0.14^cd^	9.35 ± 0.01^b^	12.19 ± 1.87^a^	SPME
1311	2463-77-6	Undecenal	12 ± 0.46^e^	43.08 ± 0.74^c^	38.68 ± 4.48^c^	30.63 ± 0.32^d^	36.71 ± 1.09^cd^	–	–	2.23 ± 0.09^f^	52.4 ± 3.18^b^	64.09 ± 5.31^a^	53.81 ± 1.42^b^	SPME/SAFE
1502	10,486–19-8	Tridecanal	7.89 ± 0.39^e^	7.66 ± 0.85^e^	4.4 ± 0.23^ef^	3.48 ± 0.02^ef^	17.01 ± 4.12^cd^	15.31 ± 1.4^cd^	12.63 ± 0.62^d^	18.99 ± 1.02^bc^	22.02 ± 0.50^b^	30.27 ± 0.54^a^	–	SPME/SAFE
1601	124–25-4	Tetradecanal	–	–	40.43 ± 2.52^bc^	5.4 ± 0.2^d^	24.24 ± 1.62^cd^	–	70.2 ± 7.78^a^	–	72.5 ± 8.37^a^	63.32 ± 24.65^ab^	40.01 ± 1.94^bc^	SAFE
1707	2765-11-9	Pentadecanal	8.89 ± 1.51^d^	10.33 ± 0.01^d^	11.55 ± 0.08^d^	9.55 ± 0.25^d^	12.22 ± 0.08^d^	9.24 ± 0.72^d^	23.56 ± 0.48^c^	46.5 ± 1.27^b^	47.51 ± 2.07^b^	414.99 ± 6.89^a^	–	SAFE
1800	629–80-1	1-Hexadecanal	6.75 ± 0.15^d^	9.84 ± 0.34^d^	9.67 ± 6.85^d^	5.03 ± 1.23^d^	6.39 ± 0.22^d^	3.61 ± 0.22^d^	–	46.87 ± 5.05^c^	45.49 ± 3.13^c^	88.15 ± 3.88^a^	71.82 ± 6.55^b^	SAFE
Alcohol														
671	616–25-1	1-Penten-3-ol	8.94 ± 0.22^i^	14.26 ± 0.35^fg^	31.74 ± 0.70^b^	16.26 ± 0.29^f^	21.34 ± 0.29^e^	37.78 ± 1.17^a^	8.9 ± 0.27^i^	11.13 ± 1.29^h^	24.54 ± 0.62^d^	28.74 ± 0.01^c^	13.21 ± 0.59^g^	SPME
860	111–27-3	1-Hexanol	49.06 ± 6.72^e^	381.42 ± 62.16^d^	3673.74 ± 148.08^b^	478.56 ± 9.69^d^	1897.89 ± 2.35^c^	9223.37 ± 114.82^a^	55.23 ± 4.06^e^	59.38 ± 3.07^e^	84.4 ± 1.46^e^	49.61 ± 1.09^e^	64.61 ± 2.74^e^	SPME
960	111–70-6	Heptanol	32.45 ± 0.93^g^	73.09 ± 0.85^d^	97.35 ± 0.17^b^	41.44 ± 0.63^f^	89.51 ± 5.86^c^	192.82 ± 0.11^a^	84.69 ± 0.39^c^	74.11 ± 0.61^d^	64.31 ± 0.62^e^	39.8 ± 0.04^f^	67.07 ± 1.64^e^	SPME
969	3391-86-4	1-Octen-3-ol	263.88 ± 11.06^h^	747.98 ± 19.65^d^	1006.55 ± 11.48^b^	531.11 ± 16.86^f^	786.6 ± 6.08^c^	1137.23 ± 3.66^a^	454.28 ± 9.85^g^	471.11 ± 14.46^g^	645.87 ± 0.86^e^	449.94 ± 9.12^g^	533.74 ± 16.96^f^	SPME/SAFE
1063	118–71-8	Maltol	–	–	–	–	–	–	–	28.32 ± 0.33^c^	42.3 ± 1.65^b^	187.17 ± 0.34^a^	–	SAFE
1068	35,192–73-5	1-Nonen-4-ol	27.57 ± 0.88^h^	91.31 ± 0.09^d^	252.86 ± 4.55^b^	65.12 ± 2.12^e^	183.01 ± 14.51^c^	430.53 ± 0.51^a^	48.79 ± 0.62^f^	52.34 ± 0.98^ef^	91.63 ± 2.34^d^	55.4 ± 1.58^ef^	–	SPME/SAFE
Acids														
550	64–18-6	Formic Acid	–	–	–	–	–	–	13.65 ± 0.43c	14.8 ± 1.06c	22.85 ± 1.75b	11.01 ± 0.02d	30.97 ± 1.49a	SPME
576	64–19-7	Acetic acid	11.36 ± 1.12^g^	70.37 ± 7.67^de^	99.1 ± 1.36^c^	61.84 ± 0.29^ef^	44.91 ± 1.15^f^	86.14 ± 0.68^cd^	76.47 ± 1.06^cde^	166.78 ± 19.67^b^	163.94 ± 5.19^b^	209.04 ± 7.99^a^	147.1 ± 0.9^b^	SPME
790	107–92-6	Butyric Acid	1.4 ± 0.13^c^	14.85 ± 1.54^b^	–	14.41 ± 0.29^b^	–	24.67 ± 3.81^a^	–	–	–	10.89 ± 1.79^b^	22.12 ± 2.65^a^	SPME
811	503–74-2	3-Methylbutanoic acid	0.47 ± 0.01^g^	5.46 ± 0.11^d^	2.96 ± 0.52^e^	1.66 ± 0.02^f^	10.89 ± 0.11^c^	–	1.39 ± 0.10^f^	1.78 ± 0.03^f^	20.92 ± 0.12^a^	15.91 ± 0.40^b^	–	SPME
974	142–62-1	1-Hexanoic acid	29.29 ± 4.24^de^	37.81 ± 6.48^cd^	25.73 ± 0.27^e^	47.49 ± 5.52^c^	37.23 ± 2.15^cd^	23.69 ± 2.24^e^	7.68 ± 0.2^f^	41.07 ± 0.37^c^	91.75 ± 4.07^b^	189.49 ± 0.25^a^	71.97 ± 3.63^b^	SPME/SAFE
1173	124–07-2	Octanoic acid	2.47 ± 0.09^c^	1.41 ± 0.06^c^	–	44.42 ± 1.13^b^	79.47 ± 11.01^a^	4.15 ± 0.77^c^	5.79 ± 0.51^c^	10.79 ± 0.66^c^	9.78 ± 1.83^c^	7.65 ± 0.06^c^	–	SPME/SAFE
1272	112–05-0	Nonanoic acid	3.99 ± 0.32^g^	10.12 ± 1.02^f^	11.22 ± 2.72^f^	19.67 ± 1.61^e^	10.95 ± 0.16^f^	9.62 ± 1.25^f^	8.41 ± 0.62^fg^	30.28 ± 1.97^c^	71.69 ± 1.98^a^	25.25 ± 1.40^d^	62.63 ± 1.06^b^	SPME/SAFE
Ketone														
698	116–09-6	Acetol	1.45 ± 0.47^gh^	5.47 ± 0.77^e^	3.7 ± 0.86^efg^	2.42 ± 0.01^fgh^	3.96 ± 0.39^ef^	1.15 ± 0.27^h^	5.72 ± 0.53^e^	13.65 ± 0.54^d^	40.21 ± 0.14^c^	48.42 ± 1.92^b^	64.71 ± 0.30^a^	SPME
717	513–86-0	Acetoin	76.2 ± 2.83^a^	34.86 ± 1.23^b^	9.98 ± 1.50^d^	39.34 ± 0.01^b^	69.8 ± 0.17^a^	69.69 ± 0.62^a^	43.39 ± 5.37^b^	30.32 ± 2.93^bc^	61.96 ± 12.13^a^	40.07 ± 3.68^b^	19.37 ± 1.89^cd^	SPME/SAFE
754	591–78-6	2-Hexanone	0.61 ± 0.13^cd^	0.87 ± 0.12^cd^	2.02 ± 0.19^b^	1.01 ± 0.17^c^	1.72 ± 0.02^b^	2.56 ± 0.04^a^	0.50 ± 0.12^d^	0.50 ± 0.10^d^	–	–	1.79 ± 0.28^b^	SPME
790	600–14-6	2,3-Pentanedione	–	–	–	–	–	–	–	–	31.14 ± 5.22^c^	67.92 ± 2.04^b^	106.02 ± 0.65^a^	SAFE
853	110–43-0	2-Heptanone	18.91 ± 1.05^h^	51.65 ± 0.98^c^	59.99 ± 0.01^b^	38.42 ± 1.39^e^	51.67 ± 0.98^c^	63.55 ± 0.56^b^	51.2 ± 2.62^c^	46.39 ± 1.4^d^	29.98 ± 0.48^f^	71.64 ± 0.70^a^	24.35 ± 0.45^g^	SPME
943	4312-99-6	1-Octen-3-one	1.81 ± 0.08^g^	4.31 ± 0.15^fg^	6.56 ± 0.27^ef^	3.26 ± 0.13^g^	3.69 ± 0.02^g^	6.45 ± 0.16^ef^	8.64 ± 0.16^de^	14.75 ± 1.51^b^	21.51 ± 1.97^a^	11.8 ± 0.63^c^	10.48 ± 0.10^cd^	SPME/SAFE
960	1669-44-9	3-Octen-2-one	3.18 ± 0.08^e^	9.47 ± 0.21^d^	20.76 ± 1.65^a^	9.2- ± 0.10^d^	13.88 ± 0.85^bc^	16.01 ± 0.20^b^	12.39 ± 1.25^cd^	12.06 ± 0.78^cd^	15.09 ± 1.72^bc^	12.11 ± 0.82^cd^	16.79 ± 1.39^b^	SPME
988	923–28-4	2-Methyl-3-octanone	14.93 ± 0.04^e^	62.48 ± 1.98^c^	83.28 ± 3.93^b^	63.89 ± 2.54^c^	67.18 ± 3.46^c^	95.51 ± 2.31^a^	67.99 ± 0.46^c^	47.3 ± 0.05^d^	61.61 ± 4.55^c^	–	–	SPME
1029	98–86-2	Acetophenone	1.78 ± 0.01^d^	2.49 ± 0.20^d^	1.84 ± 0.07^d^	5.02 ± 0.01^d^	5.47 ± 0.44^d^	2.52 ± 0.13^d^	5.99 ± 1.29^d^	30.76 ± 0.65^c^	30.86 ± 0.87^c^	58.34 ± 4.77^b^	149.73 ± 1.83^a^	SPME
1151	693–54-9	2-Decanone	0.11 ± 0.01^f^	1.93 ± 0.06^de^	2.79 ± 1.01^cd^	3.44 ± 0.05^bc^	2.03 ± 0.14^de^	1.58 ± 0.02^de^	1.86 ± 0.03^de^	0.96 ± 0.13^ef^	4.63 ± 0.9^ab^	5.06 ± 0.35^a^	–	SPME
Furans														
727	67–71-0	Dimethyl sulfone	380.16 ± 16.09^de^	571.72 ± 15.94^c^	214.04 ± 3.88^g^	104.41 ± 1.41^h^	459.58 ± 5.05^d^	286.06 ± 8.53^fg^	306.8 ± 1.84^ef^	945.99 ± 9.81^b^	205.95 ± 4.04^g^	1080.45 ± 63.78^a^	613.58 ± 58.14^c^	SPME/SAFE
761	1487-18-9	2-Ethenylfuran	–	–	–	–	–	–	–	2.46 ± 0.31^d^	4.73 ± 0.01^c^	5.53 ± 0.34^b^	16.53 ± 0.51^a^	SPME
831	498–60-2	3-Furaldehyde	–	–	–	–	–	–	–	26,701.62 ± 1077.10a	17,195.32 ± 789.20c	14,561.16 ± 826.60d	24,269.63 ± 575.05b	SPME/SAFE
848	98–01-1	Furfural	9.25 ± 0.68^f^	17.16 ± 0.09^f^	13.17 ± 0.12^f^	8.42 ± 0.42^f^	9.92 ± 0.44^f^	–	5479.3 ± 143.95^e^	31,386.36 ± 1347.90^d^	35,566.36 ± 1559.92^c^	43,777.87 ± 1953.01^b^	49,080.21 ± 456.64^a^	SPME/SAFE
868	591–11-7	5-Methyl-2(5H)-furanone	–	–	–	–	–	–	61.34 ± 0.71^d^	860.97 ± 5.29^c^	987.45 ± 15.71^c^	1981.64 ± 91.4^b^	4242.78 ± 99.34^a^	SPME/SAFE
878	1192–62-7	1-(2-Furanyl)-ethanone	–	–	–	–	–	–	83.81 ± 3.79^e^	638.1 ± 6.75^d^	983.8 ± 144.4^c^	2580.81 ± 139.1^b^	4913.71 ± 58.03^a^	SPME/SAFE
885	98–00-0	2-Furanmethanol	–	–	–	–	–	–	39.21 ± 3.65^e^	160.57 ± 2.73^d^	389.37 ± 62.24^c^	690.72 ± 22.05^b^	809.42 ± 26.26^a^	SPME/SAFE
897	591–12-8	5-Methyl-2(3H)-furanone	–	–	–	–	–	–	32.43 ± 1.12^c^	88.78 ± 1.10^b^	104.44 ± 21.38^b^	194.26 ± 13.59^a^	199.11 ± 1.14^a^	SPME/SAFE
909	611–13-2	Methyl 2-furoate	–	–	–	–	–	–	2.14 ± 0.05^e^	321.9 ± 0.91^d^	829.39 ± 2.14^b^	427.98 ± 0.07^c^	1245.03 ± 44.16^a^	SPME
920	620–02-0	5-Methylfurfural	–	–	–	–	–	–	255.55 ± 3.36^e^	2546.34 ± 35.99^d^	2811.59 ± 60.43^c^	8129.22 ± 48.85^b^	9223.37 ± 151.61^a^	SPME/SAFE
941	4466–24-4	2-Butylfuran	–	–	–	–	–	–	–	3.57 ± 0.33^c^	7.4 ± 0.57^b^	30.34 ± 0.75^a^	–	SAFE
967	1193-79-9	2-Acetyl-5-methylfuran	–	–	–	–	–	–	–	14.34 ± 0.39^d^	23.02 ± 0.66^c^	128.82 ± 3.76^b^	512.86 ± 5.71^a^	SPME/SAFE
1006	13,493–97-5	Furfuryl formate	–	–	–	–	–	–	–	40.06 ± 0.98^b^	51.26 ± 0.08^a^	37.54 ± 1.55^b^	39.1 ± 2.11^b^	SPME
1018	271–89-6	Benzofuran	–	–	–	–	–	–	–	–	–	65.86 ± 1.56b	130.88 ± 3.98a	SPME/SAFE
1036	59,303–05-8	5-Methyl-2-furylmethanethiol	–	–	–	–	–	–	–	26.18 ± 0.90^c^	29.5 ± 0.85^b^	52.67 ± 0.78^a^	–	SPME
1038	623–30-3	3-(2-Furanyl)-2-propenal	–	–	–	–	–	–	10.68 ± 0.45^e^	133.88 ± 5.69^d^	320.7 ± 17.31^b^	261.1 ± 4.80^c^	624.86 ± 21.23^a^	SPME
1065	5905–00-0	2,2’-Bifuran	–	–	–	–	–	–	17.1 ± 0.61^d^	59.24 ± 5.64^c^	100.98 ± 1.72^b^	98.44 ± 0.11^b^	144.76 ± 4.76^a^	SPME/SAFE
1109	823–82-5	2,5-Furandicarboxaldehyde	–	–	–	–	–	–	20.09 ± 0.39^b^	152.72 ± 5.65^a^	–	156.99 ± 5.48^a^	–	SAFE
1121	17,678–19-2	Hydroxymethylfuran ketone	–	–	–	–	–	–	320.4 ± 4.47^d^	2772.2 ± 21.23^c^	3120.36 ± 55.89^c^	6269.52 ± 277.7^b^	8507.55 ± 310.18^a^	SPME/SAFE
1163	67–47-0	5-Hydroxymethylfurfural	–	–	–	–	–	–	936.52 ± 17.16^c^	12,132.21 ± 842.20^b^	14,247 ± 966.35^b^	21,776.32 ± 3558.26^a^	22,154.63 ± 3376.36^a^	SPME/SAFE
1509	4349-14-8	5-Furfurylhydantoin	–	–	–	–	–	–	2.1 ± 0.43^d^	8.13 ± 1.69^d^	252.33 ± 10.31^c^	623.74 ± 34.57^b^	862.62 ± 103.50^a^	SAFE
1442	33,488–56-1	5-(2-Furanylmethyl)-2-furaldehyde	–	–	–	–	–	–	–	129.95 ± 6.3^c^	194.58 ± 0.36^b^	88.91 ± 0.43^d^	369.9 ± 11.56^a^	SPME
Esters														
882	638–49-3	Pentyl formate	6.67 ± 0.26^c^	16.2 ± 1.52^a^	17.94 ± 1.86^a^	7.3 ± 0.79^c^	13.73 ± 0.05^b^	11.81 ± 0.02^b^	–	2.04 ± 0.11^d^	–	–	–	SPME
974	3050-69-9	Vinyl hexanoate	1.66 ± 0.09^ef^	5.91 ± 0.54^c^	4.88 ± 0.33^cd^	2.95 ± 0.31^de^	70.23 ± 0.91^a^	4.74 ± 0.42^cd^	5.26 ± 0.85^c^	4.96 ± 0.16^cd^	–	–	68.01 ± 1.41^b^	SPME
981	629–33-4	Hexyl formate	1.11 ± 0.13^e^	4.05 ± 0.13^c^	4.68 ± 0.21^b^	2.49 ± 0.29^d^	–	13.1 ± 0.15^a^	1.04 ± 0.13^e^	–	–	–	–	SPME
Hydrocarbons														
815	1002-33-1	1,3-Octadiene	3.21 ± 0.48^d^	6.4 ± 1.17^c^	9.58 ± 0.54^a^	5.98 ± 0.73^c^	7.53 ± 0.12^bc^	7.98 ± 0.21^b^	–	3.41 ± 0.30^d^	–	–	–	SPME
1214	112–40-3	Dodecane	11.26 ± 1.34^d^	10.29 ± 1.03^d^	8.46 ± 0.40^d^	9.25 ± 0.25^d^	18.39 ± 0.62^c^	17.85 ± 2.06^c^	17.46 ± 5.04^c^	22.91 ± 1.16^c^	35.25 ± 0.60^b^	91.5 ± 1.87^a^	–	SPME/SAFE
1313	629–50-5	Tridecane	15.84 ± 0.94^ab^	16.33 ± 3.21^ab^	5.71 ± 1.00^bc^	6.57 ± 1.26^bc^	12.59 ± 0.40^bc^	11.22 ± 11.22^bc^	9.75 ± 0.79^bc^	14.98 ± 2.15^ab^	26.69 ± 1.49^a^	–	–	SPME/SAFE
1385	61,141–72-8	4,6-Dimethyldodecane	3.3 ± 0.25^abc^	5.25 ± 0.87^abc^	0.92 ± 0.02^c^	2.63 ± 0.08^bc^	6.7 ± 1.17^ab^	9.03 ± 0.10^a^	7.88 ± 5.10^ab^	5.44 ± 0.25^abc^	7.05 ± 1.58^ab^	–	–	SAFE
1448	25,117–32-2	5-Methyltetradecane	1.47 ± 0.01^d^	4.53 ± 0.42^d^	14.32 ± 2.18^cd^	1.25 ± 0.12^d^	20.4 ± 7.74^c^	15.01 ± 0.86^cd^	13.13 ± 4.06^cd^	13.63 ± 8.06^cd^	22.62 ± 0.42^bc^	34.81 ± 3.66^ab^	42.39 ± 5.98^a^	SPME
1512	629–62-9	Pentadecane	3.28 ± 0.01^f^	4.52 ± 0.13^f^	–	5.89 ± 0.26^de^	13.02 ± 0.73^d^	8.05 ± 0.05^e^	14.02 ± 0.71^cd^	6.07 ± 0.41^de^	29.54 ± 0.24^a^	20.41 ± 1.81^b^	16.37 ± 1.96^c^	SAFE
1647	1560-92-5	2-Methylhexadecane	1.81 ± 0.23^cd^	12.13 ± 0.37^a^	–	–	4.68 ± 0.04^bcd^	1.54 ± 0.21^cd^	7.15 ± 1.99^abc^	–	11.17 ± 4.85^ab^	–	8.69 ± 3.78^ab^	SAFE
1711	629–78-7	Heptadecane	5.49 ± 0.02^cd^	6.25 ± 0.16^cd^	5.53 ± 0.44^cd^	1.01 ± 0.18^d^	6.01 ± 0.39^cd^	2.14 ± 0.48^d^	9.86 ± 0.70^c^	2.79 ± 0.06^cd^	50.63 ± 0.17^b^	65.94 ± 2.98^a^	67.81 ± 6.52^a^	SAFE
1746	6418-44-6	3-Methylheptadecane	6.71 ± 0.82^fg^	9.28 ± 0.52^efg^	1.85 ± 0.36^g^	20.16 ± 4.10^def^	27.89 ± 1.78^d^	42.2 ± 0.27^c^	12.66 ± 1.01^defg^	23.57 ± 11.52^de^	24.85 ± 2.97^d^	59.6 ± 7.08^b^	87.51 ± 3.60^a^	SAFE
1846	25,117–35-5	5-Methyloctadecane	1.67 ± 0.01^c^	6.25 ± 5.22^c^	2.1 ± 0.13^c^	0.54 ± 0.23^c^	1.24 ± 0.10^c^	3.03 ± 0.63^c^	3.25 ± 0.30^c^	44.4 ± 3.74^a^	2.9 ± 0.04^c^	15.45 ± 1.06^b^	–	SAFE
2009	112–95-8	Icosane	36.75 ± 4.57^d^	11.63 ± 0.35^f^	15.85 ± 0.97^ef^	11.48 ± 0.91^f^	22.86 ± 0.95^e^	13.59 ± 1.22^ef^	14.76 ± 1.75^ef^	49.36 ± 4.92^c^	59.3 ± 3.17^b^	69.56 ± 5.99^a^	13.84 ± 2.84^ef^	SAFE

Note: Different letters indicate significant differences between smoked chicken thighs with different smoking times (*P* < 0.05).

### 3.2 Changes in aroma compound concentrations in smoked chickens during the sugar-smoking process

**Fig. 1:**
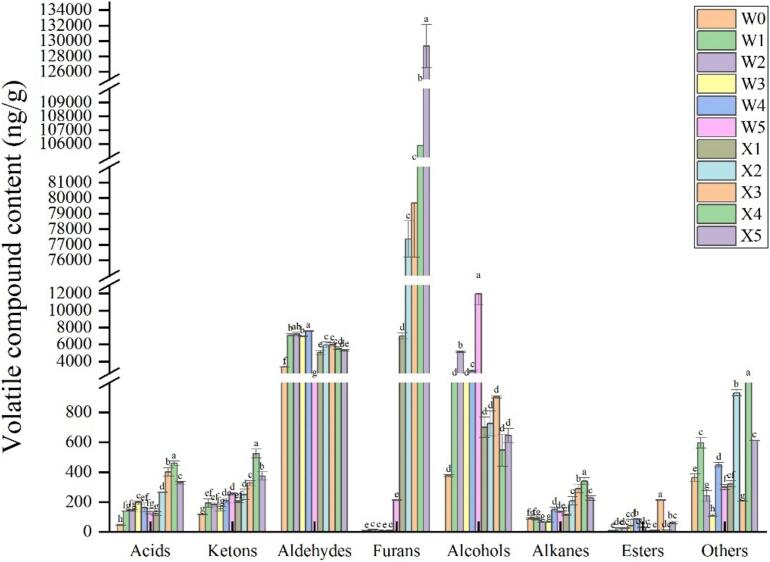
Changes in the content of various volatile flavor compounds in smoked chicken thighs during the smoking process. Different letters indicate significant differences between smoked chicken thighs with different smoking times (*P* < 0.05).

**Fig. 2:**
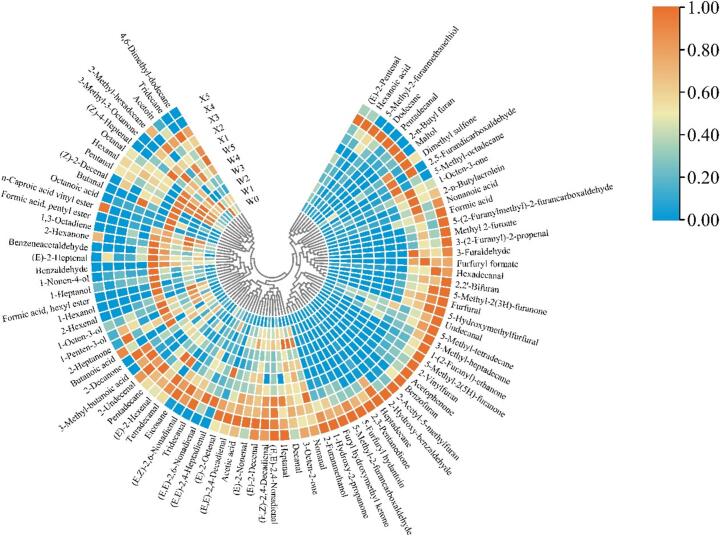
Hot plot of volatile flavor compounds content in sugar-smoked chicken thighs during the smoking process.

The detection of 91 volatile chemicals across the two extraction procedures is illustrated in Fig. 1. These compounds included 7 acids, 10 ketones, 32 aldehydes, 21 furans, 6 alcohols, 3 esters, and 12 hydrocarbons. The dynamic change in the content of volatile compounds in smoked chickens with increasing heating time is illustrated in Fig. 2. These findings showed that, relative to the contents of other types of compounds, the content of furan compounds in smoked chickens was the highest, followed by the aldehyde content.

Furans are formed by sugar cleavage reactions and the Maillard reaction. These compounds are important heterocyclic compounds that contribute to the flavor of smoked chickens. As shown in Table 1, unlike in the sugar-smoked group, only two furans compounds, dimethyl sulfone and furfural, were detected in the group without smoking material, indicating that sugar smoking promoted the formation of furans, which was similar to the findings of Chang et al. (2021). Furfural is an important furan compound found in smoked chickens, its content is the highest, and the threshold is low. The furfural content after heating for 1 min was greater than the content after heating for 2–5 min. In the early stage of pyrolysis, sucrose mainly forms intermediates such as glucose and fructose through dehydration reactions. These intermediate products are further degraded and cyclized to form furfural. With increasing heating time, furfural may polymerize or react with other decomposition products to form other compounds, thus reducing the furfural content, and penetrate smoked chickens through flue gas adsorption to give the smoked chicken a caramel flavor and a toasty aroma. In this study, the amount of 5-hydroxymethylfurfural (5-HMF) significantly increased as the heating time increased. This occurred probably because sucrose was not properly broken down, resulting in the formation of more active dicarbonyl compounds that directly or indirectly affected the formation of 5-HMF in smoked chickens. Wang et al. (2022) reported that furfural and 5-HMF contributed the most to the smoky aroma of smoked chickens. The compounds 5-methyl-2(5H)-furanone, 1-(2-furyl)-ethyl ketone, 3-furfural, 2-furanmethanol, 5-methylfurfural, 2,5-furandiformal, 2-acetyl-5-methylfuran, and 5-methyl-2(3H)-furanone are the late products of sucrose pyrolysis. They impart a rich caramel, roasting, and smoking aroma to smoked chickens, and their content increases significantly as the smoking time increases. Thus, smoking time plays a role in enhancing and balancing the aroma of smoked chickens. In smoked chickens, the most abundant volatile compounds are aldehydes, which are produced mainly through lipid oxidative degradation and the Strecker reaction (Zamora et al., 2015). Five dienal compounds, namely, (*E*,*E*)-2,6-nonadienal, (*E*,*Z*)-2,6-nonadienal, (*E*,*E*)-2,4-nonadienal, (*E*,*Z*)-2,4-decadienal, and (*E*,*E*)-2,4-decadienal, which impart fat aroma and meat flavor to smoked chickens, were detected in smoked chickens. The content of (*E*,*E*)-2,4-decadienal reached 114.17 ng/g at 4 min of sugar fumigation, which was about twice the content recorded in the group without smoking material, indicating that the pyrolysis of sucrose favored the formation of such compounds. Linoleic acid is formed by a radical chain reaction to hydroperoxide, followed by a further *β*-shear reaction to form (*E*,*E*)-2,4-decadienal. (*E*,*E*)-2,4-decadienal has a very low aroma threshold of 0.027 ng/g, indicating that it can impart a significant fatty flavor to meat products even at very low concentrations. This phenomenon has been confirmed in various products, such as roasted lambs and braized lambs (Wu et al., 2023). Only three ester compounds, pentyl formate, hexyl formate, and vinyl n-caproate, are found in smoked chickens, and they exhibit fruity and floral aromas. Pentyl formate was detected mainly in the group without smoking material and was detected after heating for 2 min in the sugar-smoked group. In this group, the content reached a peak of 17.94 ng/g within 2 min of heating and decreased gradually to 11.81 ng/g as the heating time increased. This phenomenon may be attributed to the accelerated reaction rate of precursor compounds during the initial heating phase, where the elevated temperature facilitates the rapid accumulation of pentyl formate. However, as the heating time extends, high temperatures may trigger the thermal decomposition or volatilization of ester compounds, while the gradual consumption of precursor substances (such as acids and alcohols) leads to a decrease in the formation rate, ultimately resulting in a decline in pentyl formate concentration. Hexyl formate reached its maximum value of 13.10 ng/g in the group without smoking material after 5 min of heating, whereas it was detected only after heating for 1 min in the sugar-smoked group. These results suggested that the sugar fumigation process may inhibit the formation of ester compounds or that the sucrose pyrolysis product may promote the degradation of ester compounds.

### 3.3 Changes in free radicals in smoked chickens during the sugar-smoking process

Common processing methods in food, such as irradiation, heating, and high pressure, can lead to the formation of free radicals (Andersen et al., 2011; Bolumar et al., 2014; Escudero et al., 2012). Free radicals are produced from many sources, such as proteins (including structural proteins and water-soluble proteins), lipids (mainly unsaturated fatty acids), carbohydrates, and soluble vitamins, which are components of food. Heat can promote lipid oxidation in meat products, and unsaturated fatty acids are easily induced by free radicals to undergo lipid peroxidation, which is an important intermediate product of lipid oxidation. Therefore, changes in the contents of hydroxyl radicals and lipid radicals (alkyl peroxy radicals, alkyl radicals and alkoxy radicals) in smoked chickens were detected during the 5 min of smoking.

**Fig. 3:**
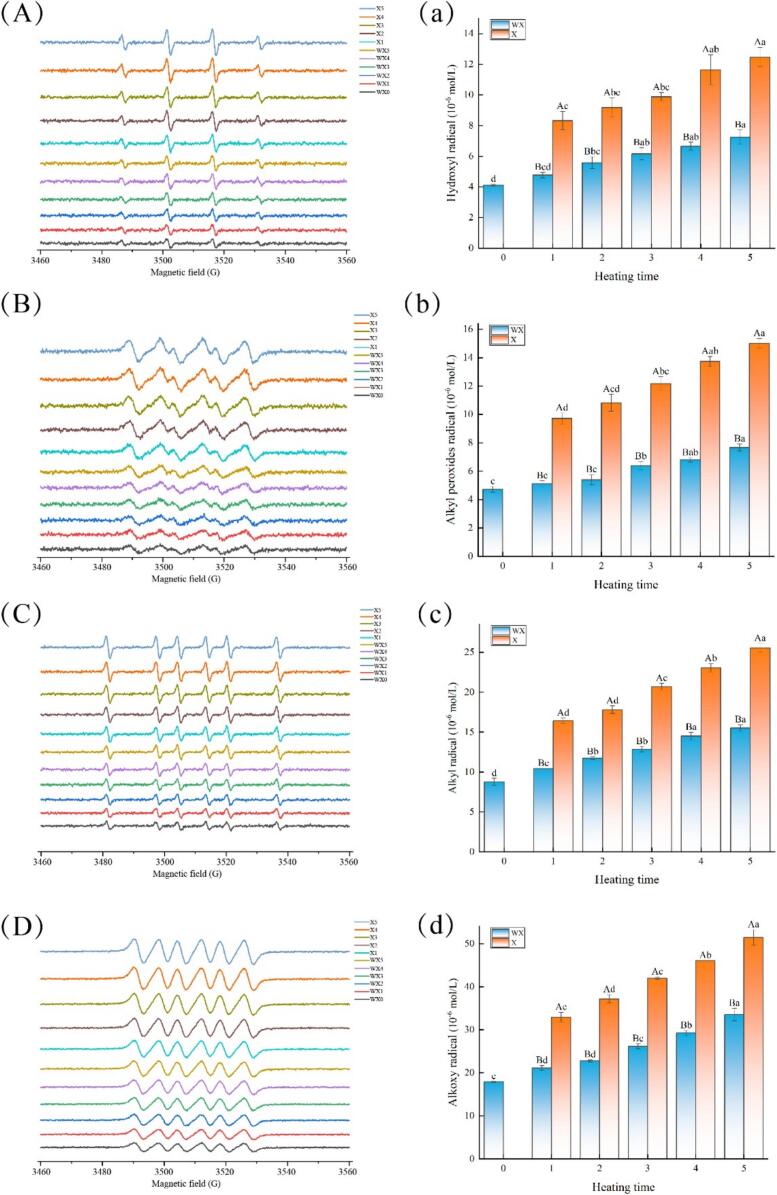
The ESR spectra of free radicals in smoked chicken thighs during the smoking process (A: Hydroxyl radical; B: Alkyl peroxy radicals; C: Alkyl radicals; D: Alkoxy radicals); The variation in free radical content in smoked chicken thighs during the smoking process (a-d). “WX” represents the group without smoking material, and “X” represents the sugar-smoked group. Lower case letters indicate significant differences between smoked chicken thighs with different smoking times, and upper-case letters indicate significant differences between different sugar-smoked groups with the same smoking time (*P* < 0.05).

The contents of the four free radicals increased as the heating time increased during the smoking process (Fig. 3), which matches the results of previous studies. Cheng et al. (2017) measured the free radical content of high-fat milk powder stored at 70 °C for 1 h and 20 days and at 25 °C for 18 weeks. They reported that the higher the storage temperature was and the longer the storage time was, the greater the free radical content in the milk powder. Yeretzian et al. (2012) reported that the content of free radicals in coffee beans roasted at 248 °C for 840 s was significantly greater than that in coffee beans roasted for 500 s. This occurred probably because, at the beginning of the smoke treatment, the rate of free radical generation in the initiation and transfer phases of lipid oxidation was greater than that in the termination phase. Thus, more lipid radicals were captured by DMPO, which increased the content of free radicals. The content of free radicals in the sugar-smoked group was significantly greater than that in the non-smoked group, indicating that sugar smoking favored the generation of free radicals. The ESR results of the sugar smoking group at 5 min revealed that the contents of various radicals decreased in the following order: alkoxy radicals (51.43 × 10^−6^ mol/L) > alkyl radicals (25.56 × 10^−6^ mol/L) > alkyl peroxy radicals (15.01 × 10^−6^ mol/L) > hydroxyl radicals (12.47 × 10^−6^ mol/L). Through kinetic analysis of the lipid oxidation process, Labuza and Dugan (1971) reported that the decomposition products of alkyl peroxy radicals and alkoxy radicals have high reactivity and are the initiators of automatic catalytic oxidation initiation. Hydroxyl radicals are oxygen radicals with oxygen atoms at their core. They have extremely high activity, short half-lives, and instability. Thus, they can be detected at relatively low levels. Among all oxygen radicals, hydroxyl radicals cause the greatest damage. During lipid oxidation, alkyl radicals are the first lipid radicals to be produced. Alkoxy radicals are intermediate products of lipid oxidation and have a longer lifespan than hydroxyl radicals, they can diffuse in organisms and attack other macromolecules. Alkyl peroxy radicals are also among the main intermediates of lipid oxidation, but their activity is lower than that of alkoxy radicals. After alkane peroxy radicals are produced, they first form a free radical centered on carbon atoms through cyclization. On the basis of the highest content of alkoxy radicals in smoked chickens and the activity of these radicals, it is speculated that alkoxy radicals are the most important free radicals for lipid oxidative degradation in smoked chickens.

### 3.4 Correlation analysis

OAVs are important indicators for measuring the contribution of aroma compounds to the overall aroma. The concentration of volatile flavor compounds in smoked chicken thighs was preliminarily quantified using the internal standard method. Based on this, volatile flavor compounds with an OAVs >1 were used the calibration equations (R^2^ > 0.95), with good linear regression coefficients (Table 3). As shown in Table 4, a total of 22 key aroma compounds with OAVs >1 were detected. The odor thresholds of each compound were referenced from Van Gemert (2003).

**Table 3:** Calibration equations for volatile flavor compounds of OAVs >1.

Name	Calibration equation	R^2^
Butanal	y = 0.0006× + 13.21	0.9926
Pentanal	y = 0.00003× + 2.3	0.9814
Hexanal	y = 0.00001× + 25.8	0.9902
Furfural	y = 0.000002×-100.618	0.9931
Heptanal	y = 0.00005× + 12.47	0.9716
1-(2-Furanyl)-ethanone	y = 0.000001× + 9.01	0.9619
5-Methyl-2-furancarboxaldehyde	y = 0.000002× + 0.06	0.9923
1-Octen-3-one	y = 0.00003×-14.07	0.9676
1-Octen-3-ol	y = 0.00002×-14.1	0.9557
Octanal	y = 0.00003× + 123.65	0.9965
(E)-2-Octenal	y = 0.00008×-130.01	0.9923
Furyl hydroxymethyl ketone	y = 0.00006× + 7.14	0.9914
Nonanal	y = 0.00002×-64.3	0.9703
(E,E)-2,6-Nonadienal	y = 0.00001×-5.81	0.9965
(E,Z)-2,6-Nonadienal	y = 0.0001× + 1.06	0.9935
(E)-2-Nonenal	y = 0.00006× + 12.2	0.9849
Decanal	y = 0.00002×-7.25	0.9934
(E,E)-2,4-Nonadienal	y = 0.001× + 0.0093	0.9759
(E)-2-Decenal	y = 0.00003× + 2.43	0.9978
Undecanal	y = 0.00002×-14.89	0.9953
(E,E)-2,4-Decadienal	y = 0.00003× + 114.2	0.9933
Tetradecanal	y = 0.0002× + 0.063	0.9834

Note: “x” represents the peak area, and “y” represents the concentration (mg/g).

**Table 4:** Volatile flavor compounds with OAVs >1 in smoked chicken thighs.

Name	CAS	Odor threshold(ng/g)	OAV										
			W0	W1	W2	W3	W4	W5	X1	X2	X3	X4	X5
Butanal	123–72-8	5	<1	17.47 ± 0.85	1.82 ± 0.41	3.84 ± 0.03	70.32 ± 1.89	–	17.32 ± 0.46	73.28 ± 4.45	–	–	–
Pentanal	110–62-3	150	1.29 ± 0.02	2.29 ± 0.06	2.47 ± 0.02	2.27 ± 0.02	2.35 ± 0.01	<1	1.2 ± 0.38	1.28 ± 0.05	1.03 ± 0.16	1.56 ± 0.10	1.46 ± 0.04
Hexanal	66–25-1	200	12.18 ± 0.27	25.46 ± 0.77	26.44 ± 1.10	26.49 ± 0.32	27.32 ± 0.88	2.46 ± 13.21	16.05 ± 29.19	17.39 ± 0.22	19.19 ± 0.12	15.5 ± 0.07	16.81 ± 0.77
Furfural	1998/1/1	3000	<1	<1	<1	<1	<1	–	1.826 ± 0.05	10.462 ± 0.45	11.855 ± 0.52	14.593 ± 0.65	16.36 ± 0.15
Heptanal	111–71-7	10	<1	<1	<1	<1	<1	<1	<1	<1	<1	<1	<1
1-(2-Furanyl)-ethanone	1192–62-7	500	–	–	–	–	–	–	<1	1.28 ± 0.01	1.97 ± 0.29	5.16 ± 0.28	9.83 ± 0.12
5-Methyl-2-furancarboxaldehyde	620–02-0	5000	–	–	–	–	–	–	<1	<1	<1	1.63 ± 0.01	1.84 ± 0.03
1-Octen-3-one	4312-99-6	0.1	603.33 ± 26.67	1436.67 ± 50.00	2186.67 ± 90.00	1086.67 ± 43.33	1230 ± 6.67	2150 ± 53.33	2880 ± 53.33	4916.67 ± 503.33	7170 ± 596.67	3933.33 ± 210.00	3493.33 ± 33.33
1-Octen-3-ol	3391-86-4	10	263.88 ± 11.06	747.98 ± 19.65	1006.55 ± 11.48	531.11 ± 16.86	786.6 ± 6.08	1137.23 ± 3.66	454.28 ± 9.85	471.11 ± 14.46	645.87 ± 0.86	449.94 ± 9.12	533.74 ± 16.96
Octanal	124–13-0	100	2.43 ± 0.05	4.6 ± 0.06	5.36 ± 0.22	4.28 ± 0.56	5.1 ± 0.17	1.35 ± 0.02	3.48 ± 0.001	3.41 ± 0.05	3.48 ± 0.04	2.73 ± 0.13	3.29 ± 0.08
(E)-2-Octenal	2548-87-0	150	<1	<1	<1	<1	<1	4.27 ± 0.0002	1.32 ± 0.10	<1	3.44 ± 0.14	4.17 ± 0.09	2.25 ± 0.14
Furyl hydroxymethyl ketone	17,678–19-2	1000	–	–	–	–	–	–	<1	2.77 ± 0.02	3.12 ± 0.06	6.27 ± 0.28	8.51 ± 0.31
Nonanal	124–19-6	260	1.76 ± 0.06	3.57 ± 0.07	4.36 ± 0.29	2.76 ± 0.10	3.46 ± 0.06	2.67 ± 0.007	3.63 ± 0.07	4.52 ± 0.26	3.55 ± 0.10	3.33 ± 0.14	3.93 ± 0.01
(E,E)-2,6-Nonadienal	17,587–33-6	1	<1	7.51 ± 1.44	–	1.81 ± 0.08	–	4.99 ± 0.22	1.08 ± 0.12	3.86 ± 0.24	10.78 ± 0.62	4.11 ± 0.70	–
(E,Z)-2,6-Nonadienal	557–48-2	0.05	50.4 ± 0.60	35.2 ± 4.80	49.2 ± 8.40	64.2 ± 16.20	56.00 ± 3.80	40.6 ± 1.60	61.6 ± 2.20	128.4 ± 9.80	226.2 ± 12.00	187.8 ± 14.00	–
(E)-2-Nonenal	18,829–56-6	0.2	106.05 ± 7.70	242.35 ± 2.50	332.9 ± 5.15	193.4 ± 3.95	197.9 ± 11.95	285.8 ± 2.60	213.9 ± 10.8	315.5 ± 1.20	306.7 ± 7.50	400.05 ± 17.85	219.25 ± 3.05
Decanal	112–31-2	5	4.17 ± 0.17	7.54 ± 0.05	10.45 ± 0.07	7.23 ± 0.24	8.55 ± 0.08	7.44 ± 0.11	7.65 ± 0.09	8.17 ± 0.10	8.88 ± 0.23	8.21 ± 0.23	6.66 ± 0.19
(E,E)-2,4-Nonadienal	5910-87-2	0.06	182.67 ± 0.50	457 ± 2.17	609.67 ± 24.00	351.83 ± 43.17	362.17 ± 3.40	391.33 ± 13.50	431.67 ± 48.67	790.17 ± 21.67	807.17 ± 10.33	788.33 ± 59.33	835.67 ± 90.00
(E)-2-Decenal	3913-81-3	1	10.96 ± 0.03	36.42 ± 1.36	36.81 ± 0.96	24.89 ± 2.08	35.5 ± 0.01	41.34 ± 0.06	40.37 ± 1.03	41.21 ± 3.14	43.99 ± 0.91	58.06 ± 0.26	52.15 ± 0.79
Undecanal	112–44-7	3	<1	1.53 ± 0.01	1.91 ± 0.41	1.86 ± 0.15	1.82 ± 0.01	1.81 ± 0.01	2.73 ± 0.31	1.28 ± 0.05	2.15 ± 0.05	3.12 ± 0.01	4.06 ± 0.62
(E,E)-2,4-Decadienal	25,152–84-5	0.027	723.7 ± 5.19	1881.11 ± 38.52	2291.85 ± 31.85	1545.19 ± 100.00	989.26 ± 18.52	1744.44 ± 57.41	1899.26 ± 54.44	2366.3 ± 59.63	3130.74 ± 125.19	4228.52 ± 32.96	2773.33 ± 31.85
Tetradecanal	124–25-4	60	–	–	<1	<1	<1	–	1.17 ± 0.13	–	1.21 ± 0.14	1.06 ± 0.41	<1

Note: “-” Not detected.

**Fig. 4:**
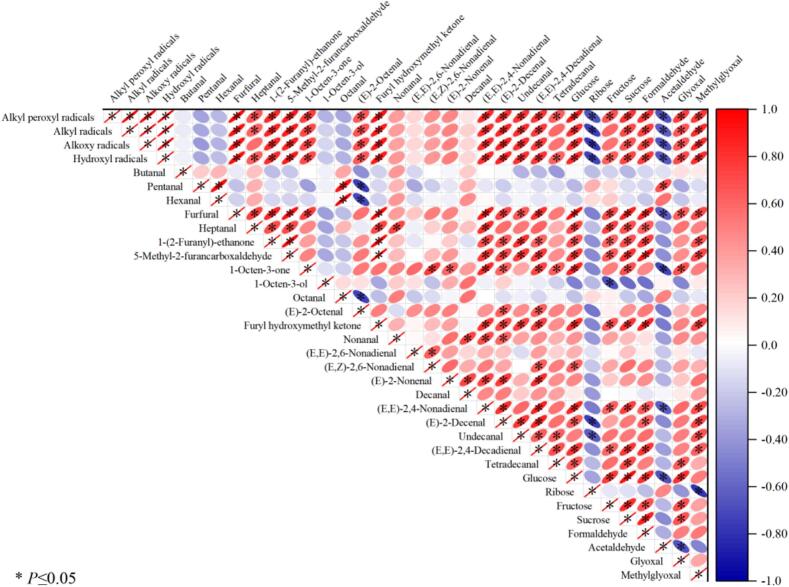
Correlation analysis of key volatile flavor compounds with reducing sugars, free radicals and carbonyl compounds in smoked chicken thighs during the smoking process.

We performed a correlation analysis of compounds with OAVs >1 in smoked chickens with sugars, free radicals, and carbonyl compounds. In Fig. 4, the red area represents a positive correlation, the blue area represents a negative correlation, and the color shading reflects the strength of the correlation. The correlation coefficients between free radicals and aroma compounds are illustrated in the color gradient shown in Fig. 4. The results of the correlation analysis revealed positive correlations between alkyl peroxy radicals, alkyl radicals, alkoxy radicals, and hydroxyl radicals, and the correlation coefficient was >0.8. Lipid oxidation typically encompasses three principal stages, namely, initiation, propagation, and termination of the free radical chain reaction, which promote lipid oxidation through mutual reactions and conversion. An increase in alkyl peroxy radicals is usually accompanied by an increase in the concentration of alkyl and alkoxy radicals (Shahidi & Zhong, 2010). Wang et al. (2022) reported that the levels of free radicals such as superoxide, hydroxyl, singlet oxygen and alkoxy radicals continued to increase as fermentation progressed. The accumulation of these free radicals accelerates the oxidation of lipids and further promotes the formation of aroma compounds. During the *β*-cleavage process, these free radicals further react with various substances to form secondary compounds, such as aldehydes, ketones, alcohols, and esters (Chang et al., 2020). As shown in Fig. 4, these radicals were positively correlated with furfural, heptanal, 1-(2-furyl)-ethyl ketone, 5-methylfurfural, 1-octen-3 one, (*E*)-2-octenal, Hydroxymethylfuran ketone, nonanal, (*E*,*E*)-2,6-nonadienal, (*E*,Z)-2,6-nonadienal, (*E*)-2-nonenal, caprical, (*E*,*E*)-2,4-nonadienal, (*E*)-2-decaenal, undecanal, (*E*,*E*)-2,4-decadienal, and tetradecanal. Free radicals were negatively correlated with butanal, pentanal, hexanal, 1-octen-3-ol, and octanal.

Free radicals play crucial roles in inducing and accelerating the oxidative degradation of unsaturated fatty acids, alcohols, sugars, and their derivatives, leading to the formation of specific flavor compounds. For example, the *β*-scission of alkoxy radicals derived from the 8th and 11th hydroperoxides of oleic acid results in the production of octanal and decanal, respectively. Similarly, the degradation of hydroperoxides of linoleic acid produces (*E*,*E*)-2,4-decadienal, hexanal, 2-pentylfuran, and 2-nonenal. Linolenic acid, owing to its greater number of double bonds, generates a greater variety of hydroperoxide isomers. For example, the 12th and 14th hydroperoxides of linolenic acid undergo *β*-scission to form (*E*)-2-pentenal and (*E*,*E*)-2,4-heptenal, respectively (Merlo et al., 2021). Frankel (1983) extensively analyzed the dynamics of alkyl and alkoxy radicals during lipid oxidation, highlighting a linear relationship between the free radical concentration and the production of nonanal and decanal. Furthermore, long-chain aldehydes such as undecanal and tetradecanal are generated via alkoxy radical decomposition during the oxidation of longer-chain unsaturated fatty acids, such as arachidonic acid (Al-Dalali et al., 2022). In thermal food processing, hydroxyl radicals significantly accelerate Maillard and carbonylation reactions involving sugars and amino acids, producing key flavor compounds such as furfural, 5-methylfurfural, 1-octen-3-one, and 5-methyl-2(5H)-furanone (Trnkova et al., 2015). Mettler et al. (2012) proposed that the glycosidic bond is broken to form a pyridyl ring with a carbonyl group at the C1 position, and then the C1—O bond is broken to open the glucopyranosyl ring. The C1 position generates an aldehyde group, the C5 position forms an alkoxy radical to attack the C2 position, the removal of OH at the C2 position forms a furan cyclic compound, the glycosidic bond at the C4 position continues to degrade, and two dehydration reactions occur to release 5-hydroxymethylfurfural. 5-Hydroxymethyl removes hydroxymethyl to form furfural, and the above pyrolysis path involves the conversion mechanism of free radicals. This suggests that free radicals not only increase the reaction rate but also influence the type and concentration of the resulting flavor compounds. However, under highly oxidative conditions, excessive free radical concentrations can lead to further degradation of flavor compounds. In highly oxidative environments, alkyl peroxyl radicals are capable of oxidizing low-molecular-weight aldehydes, such as butanal, pentanal, hexanal, and octanal, resulting in a decrease in the concentration of these volatile compounds.

Overall, these findings indicate that free radicals play a dual role in the formation and degradation of flavor compounds. Specifically, free radicals facilitate the production of key flavor compounds by promoting the oxidative degradation of unsaturated fatty acids and sugars. Conversely, excessive free radicals under highly oxidative conditions may further degrade flavor compounds, thereby compromising the flavor quality of food products. Consequently, controlling the generation and activity of free radicals during food processing is essential not only for optimizing the formation of desirable flavor compounds but also for mitigating the negative effects of over-oxidation on product quality.

## 4 Conclusion

A total of 91 volatile flavor compounds were detected in smoked chicken, among which 22 compounds exhibited OAVs >1, contributing to the characteristic roasted caramel-like, fatty, and sweet aromas of the product. A correlation analysis based on radical content was established, and the results showed that alkoxyl radicals exhibited the greatest variation, indicating that they are the most significant radical species involved in smoked chicken. For food manufacturers, the addition of carbohydrates can facilitate the formation and release of flavor compounds in meat products, thereby enhancing their overall flavor intensity. In the future study, we will explore whether the addition of a single radical species significantly affects the formation of other flavor compounds.

## CRediT authorship contribution statement

**Runmi Tao:** Writing – original draft, Resources, Methodology, Investigation, Formal analysis, Data curation. **Teng Liu:** Writing – review & editing, Validation, Methodology, Formal analysis. **Huan Liu:** Writing – review & editing, Data curation. **Jun Qi:** Writing – review & editing. **Kexin Cheng:** Software, Methodology. **Yumin Niu:** Software, Methodology. **Yuting Wang:** Resources, Investigation. **Yingying Zhang:** Software, Methodology. **Dengyong Liu:** Writing – review & editing, Supervision, Project administration, Funding acquisition, Conceptualization.

## Declaration of competing interest

The authors declare that they have no known competing financial interests or personal relationships that could have appeared to influence the work reported in this paper.

## Data Availability

Data will be made available on request.
